# Supplementary material for: Atypical functional connectome hierarchy in autism
Source: Nat Commun. 2019 Mar 4;10:1022. doi: 10.1038/s41467-019-08944-1 (PMC6399265; doi:10.1038/s41467-019-08944-1)
Supplement: Supplementary file 1 — Supplementary Information [file 41467_2019_8944_MOESM1_ESM.pdf]

Supplementary Information

Atypical functional connectome hierarchy in autism

Hong, et al.

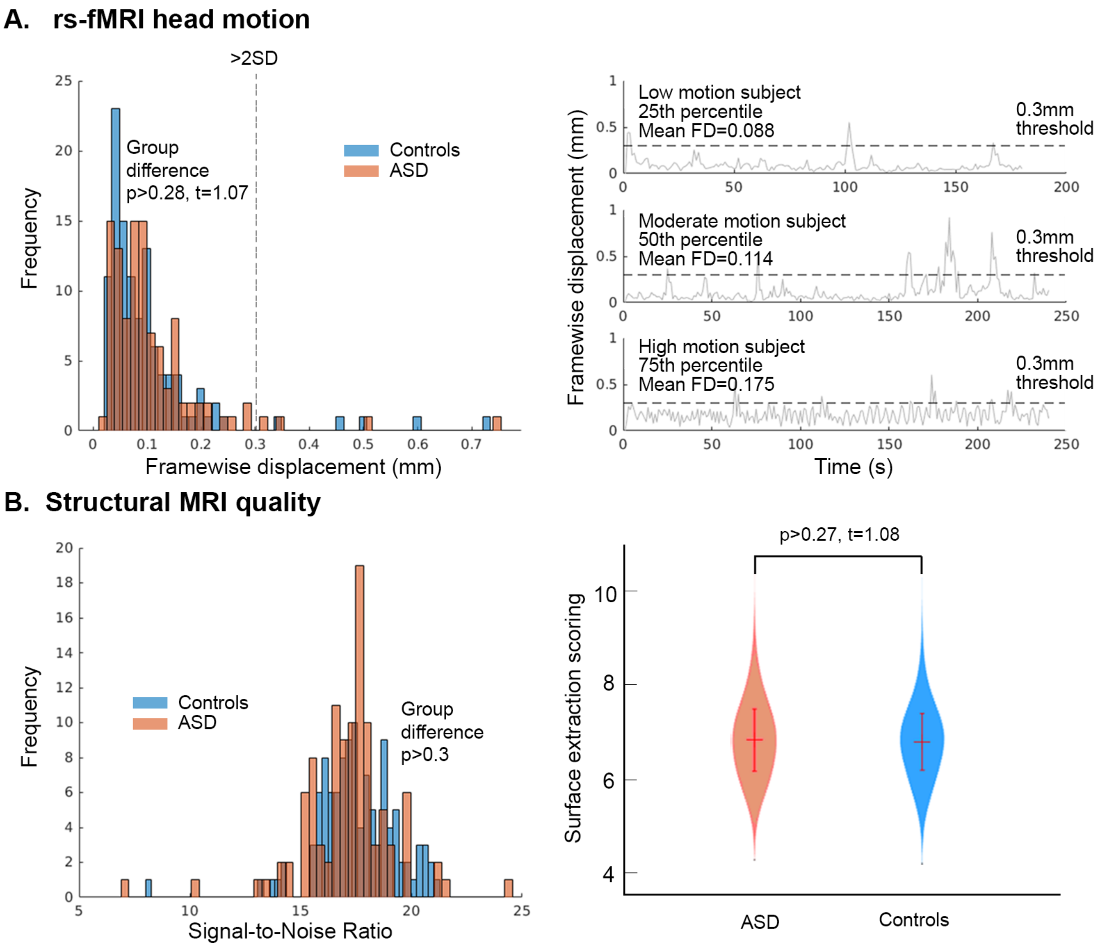

Supplementary Figure 1. A) Head motion in resting-state fMRI data. *Left*: Cumulative distribution of head motion per volume (framewise displacement [FD], in mm) for autism spectrum disorders (ASD, *orange*) and healthy controls (*blue*) in the discovery dataset. Right: Representative FD traces for low-, moderate-, and high-motion subjects at the 25th, 50th, and 75th percentiles of mean FD, respectively. Cases with FD above 0.3mm were excluded. **B)** Quality of structural MRI data used for surface extractions. *Left*: Cumulative distribution of contrast-to-noise estimates for ASD and healthy controls in the discovery dataset. *Right*: visual scoring of surface extraction. Error bars indicate 1 SD; t-statistics and p-values stem from a two-tailed Student's t-test between both groups, controlling for age and site.

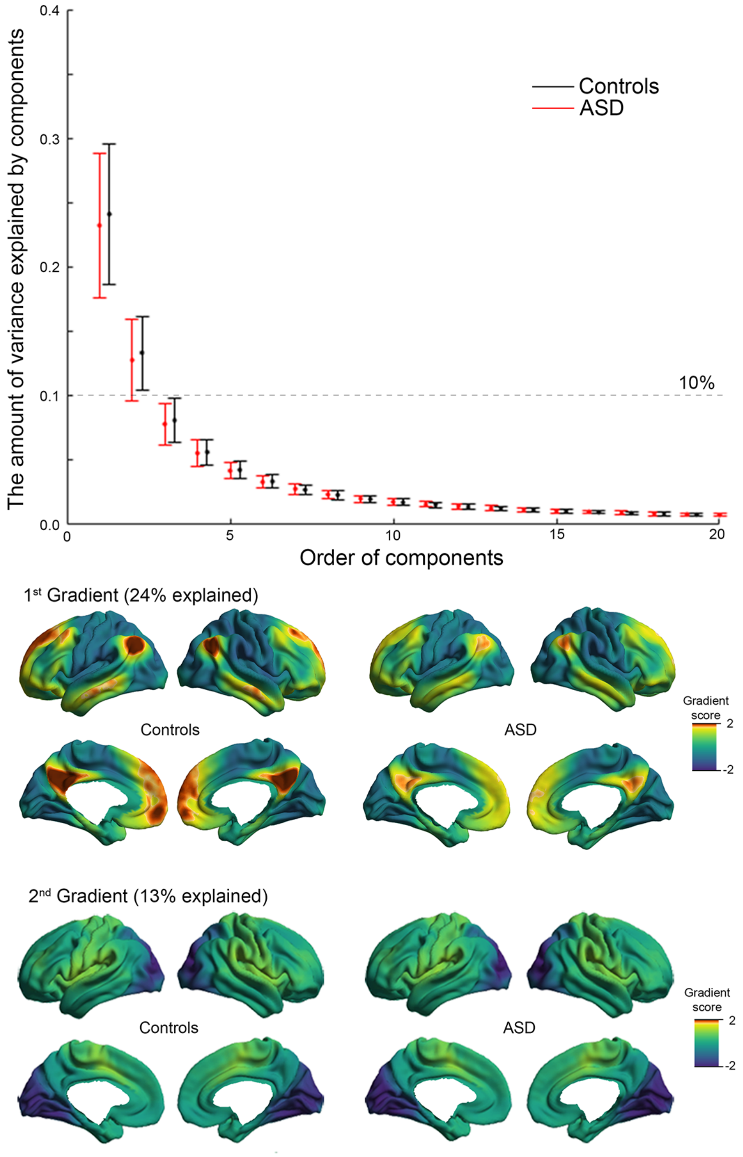

Supplementary Figure 2. *Top.* Scree plot, showing connectome-level variance explained of the first 20 components obtained from the diffusion embedding algorithm. Controls are shown in *black*, ASD in *red*. Error bars indicate 1 standard deviation. There were no differences in variance explained across the components (two-tailed Student's t-test; $p>0.1$ uncorrected). Bottom. Surface-based maps of the first two gradients.

Supra-threshold permutation test based on cluster size (10000 iterations, cluster threshold=0.01)

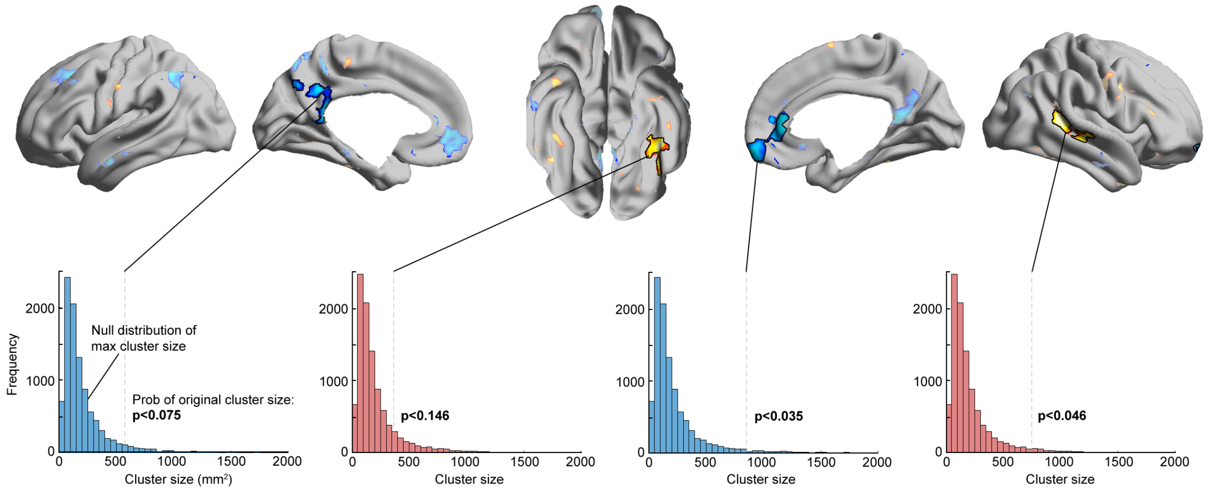

SUPPLEMENTARY FIGURE 3. Surface-based comparison of principal gradient scores between ASD and controls (see also *Figure 1*), with multiple comparisons corrected using permutation-based cluster thresholding instead of random field theory.

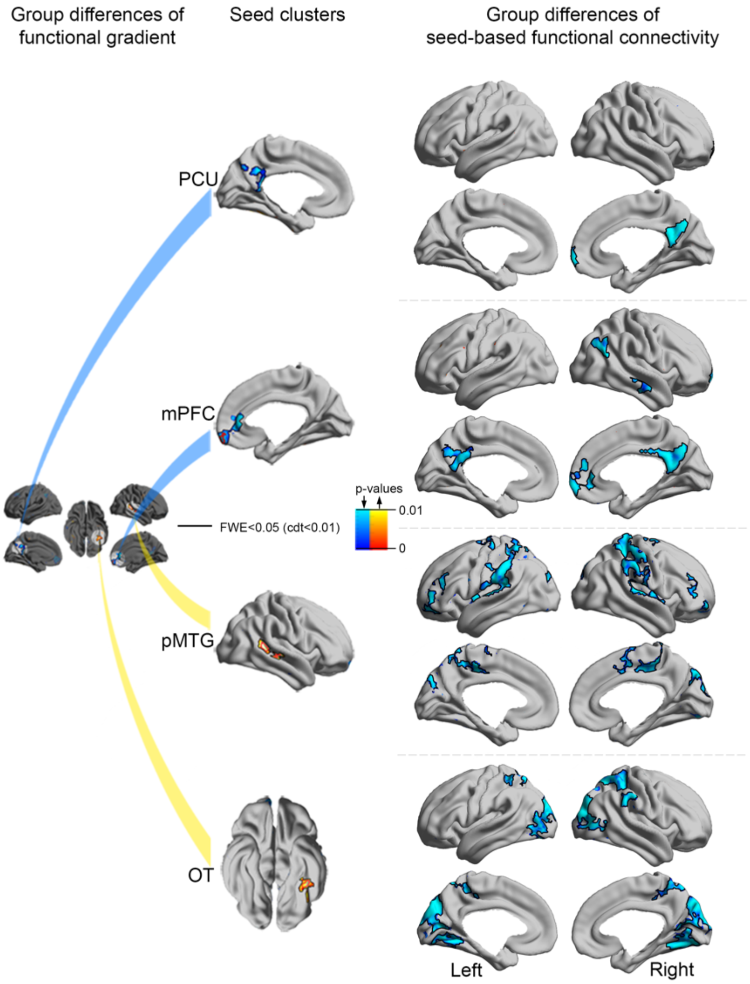

Supplementary Figure 4. Post-hoc functional connectivity analysis from significant clusters of gradient findings (see *Figure 1*), comparing ASD and controls. Connectivity decreases in ASD relative to controls are denoted in blue. No increases were significant following correction for multiple comparisons.

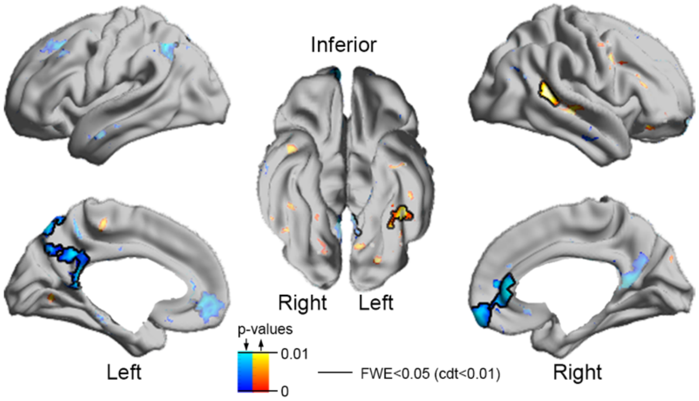

SUPPLEMENTARY FIGURE 5. Surface-based findings on differences in the first gradient, controlling for values in the second gradient. Statistical procedures are the same as for *Figure 1*.

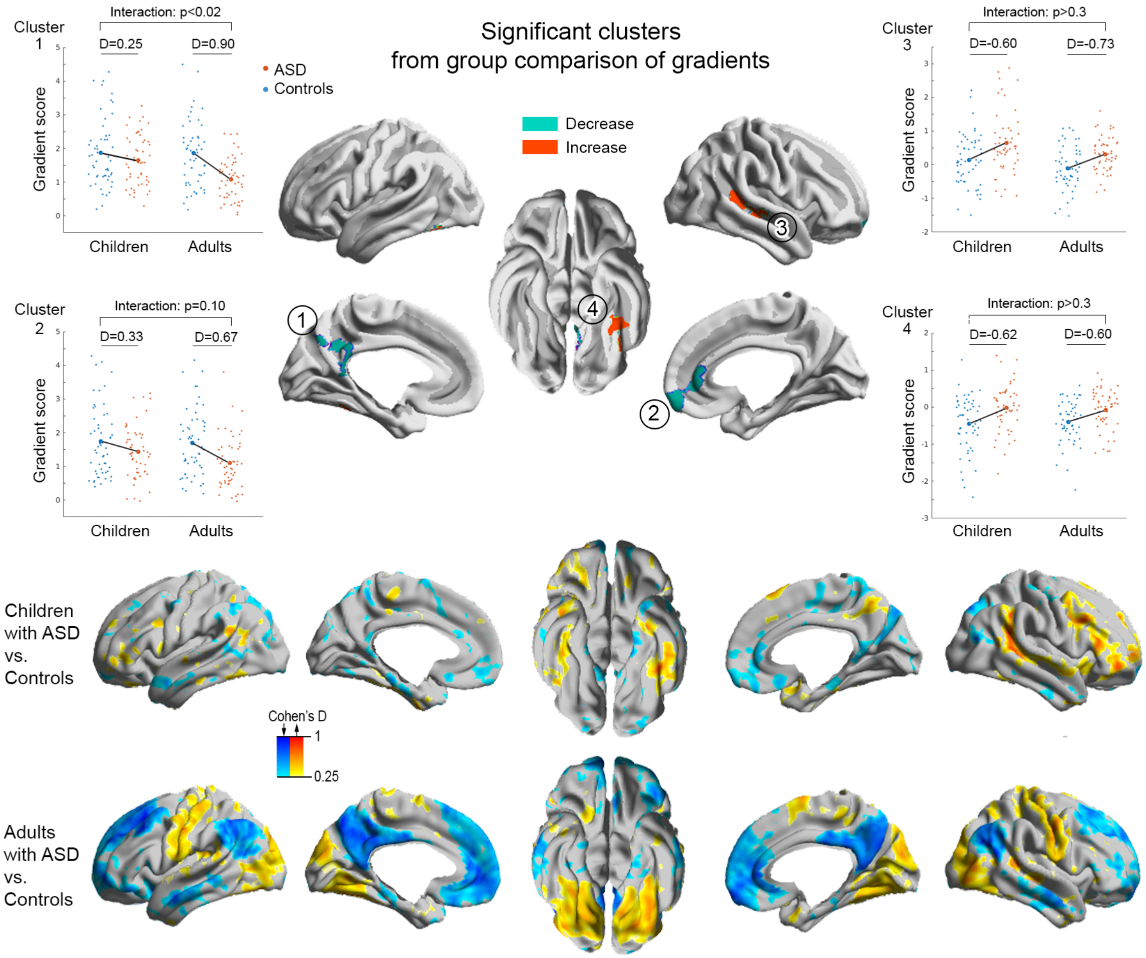

SUPPLEMENTARY FIGURE 6. Effects in children and adults. *Top.* Post-hoc gradient analysis in the discovery dataset, showing effects in adults and children, in clusters of significant findings from *Figure 1*. *Bottom.* Surface-based maps showing the effect size of group differences between ASD and controls in children and adults separately.

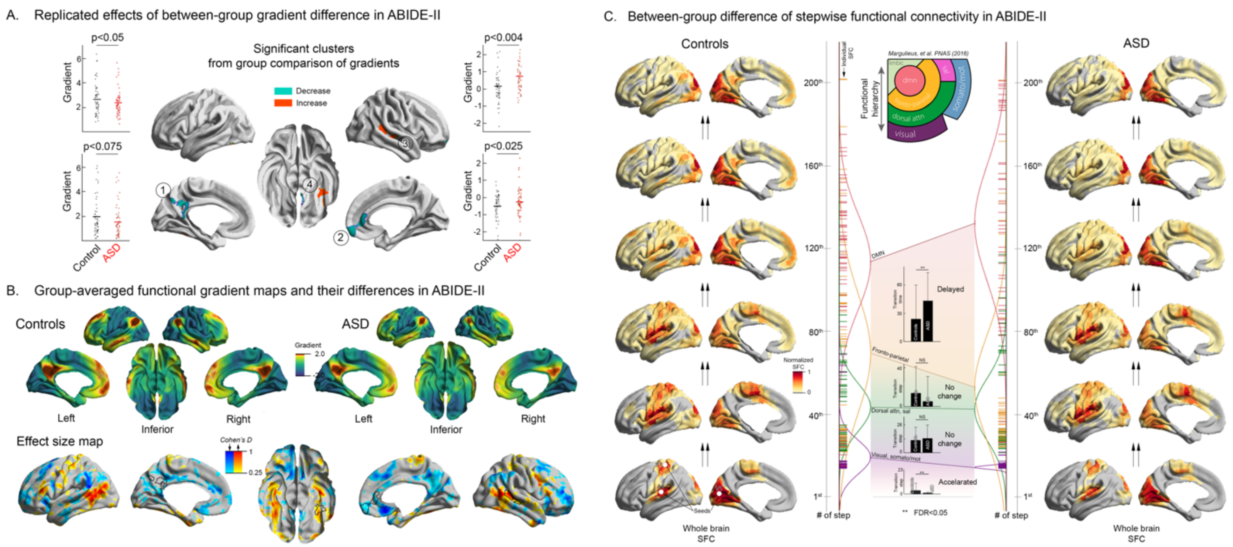

SUPPLEMENTARY FIGURE 7. Gradient and stepwise functional connectivity differences in the replication dataset. **A)** Surface-based comparison of gradient scores between controls and ASD. **B)** Effects size of differences in gradient values between ASD and controls. To evaluate the reproducibility of our original findings, significant clusters in both **A)** and **B)** were derived from the discovery dataset. **C)** Stepwise functional connectivity analysis, showing a similar disruption of propagation pattern in the replication as in the discovery cohort. For details, see *Figure 2.*

Robustness of main findings with respect to global signal regression (GSR)

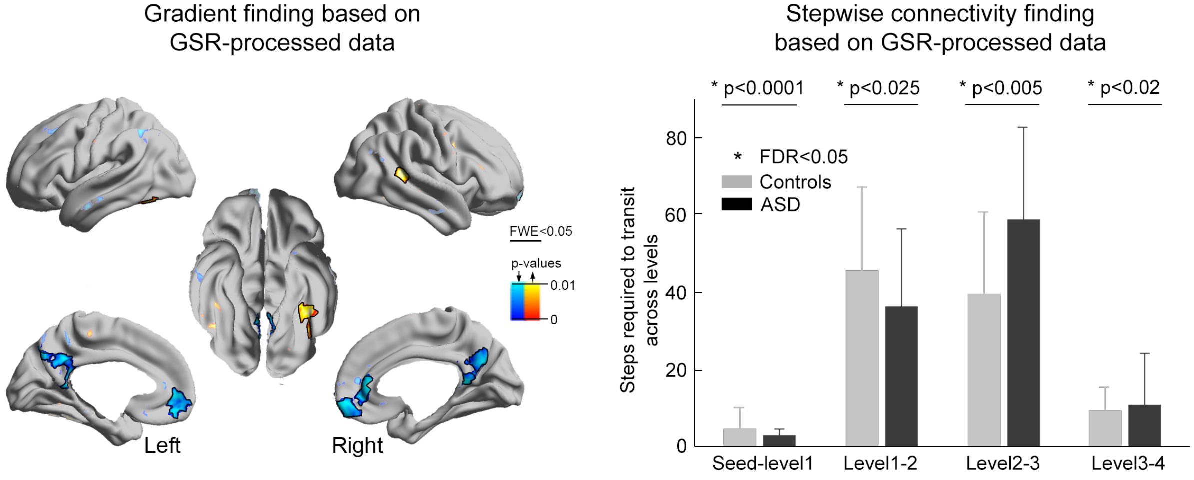

SUPPLEMENTARY FIGURE 8. Consistency of between-group differences in the first connectome gradient and stepwise functional connectivity (SFC), based on data that additionally underwent global mean signal regression (GSR). *Left.* Surface-based statistical procedures are the same as in *Figure 1*. *Right.* For SFC analysis, the different levels of hierarchy were derived from a model initially proposed by Mesulam (level 1: sensory and somatomotor networks, level 2: dorsal attention, salience networks, level 3: frontoparietal network, level 4: DMN) [37]. Comparisons are based on two-tailed Student's tests. Error bars represent 1 standard deviation.

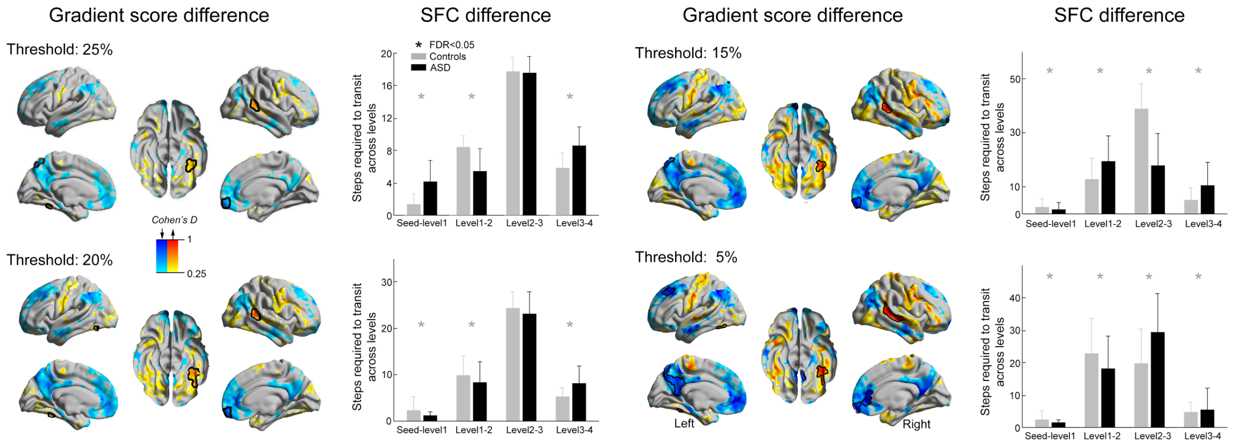

SUPPLEMENTARY FIGURE 9. Consistency of between-group differences in the first connectome gradient and stepwise functional connectivity (SFC), based on data that underwent different matrix thresholding (5-25%). Surface-based statistical procedures for gradient comparisons are as in *Figure 1.* Cohen's d effect sizes are also shown. For SFC analysis, procedures are equivalent to those in *Supplementary Figure 8*. Comparisons are based on two-tailed Student's tests. Error bars represent 1 standard deviation.

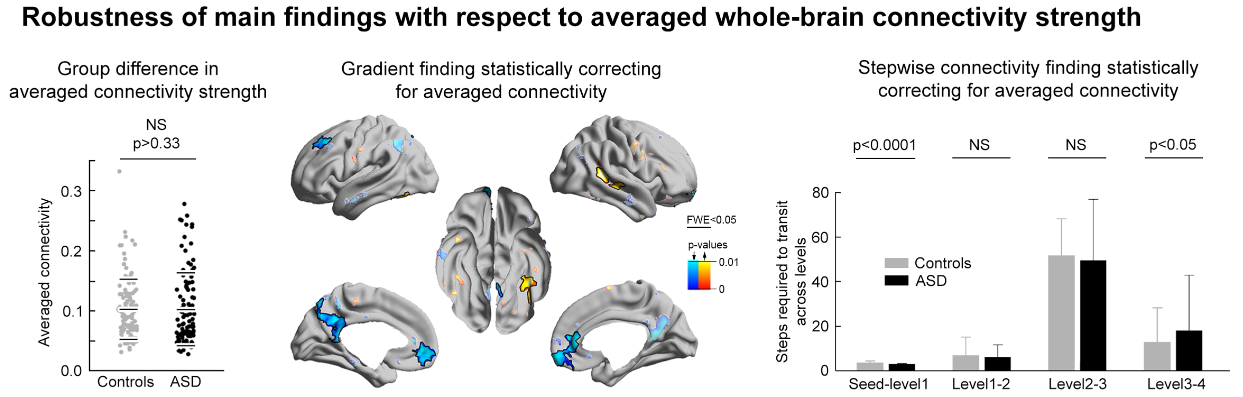

Supplementary Figure 10. Consistency of between-group differences in the first connectome gradient and stepwise functional connectivity (SFC) when additionally controlling for average connectivity shift. Left. Differences in average connectivity strength. Error bars indicate 1SD from the mean. Average connectivity strength was compared using Student's t-tests. *Middle*. Surface-based comparison in gradient when controlling for average connectivity strength. For details on statistical procedures, see *Figure 1*. *Right*. *S*FC analysis controlling for average connectivity strength. Procedures are equivalent to those in *Supplementary Figure 8*. Comparisons are based on two-tailed Student's tests. Error bars represent 1 standard deviation.

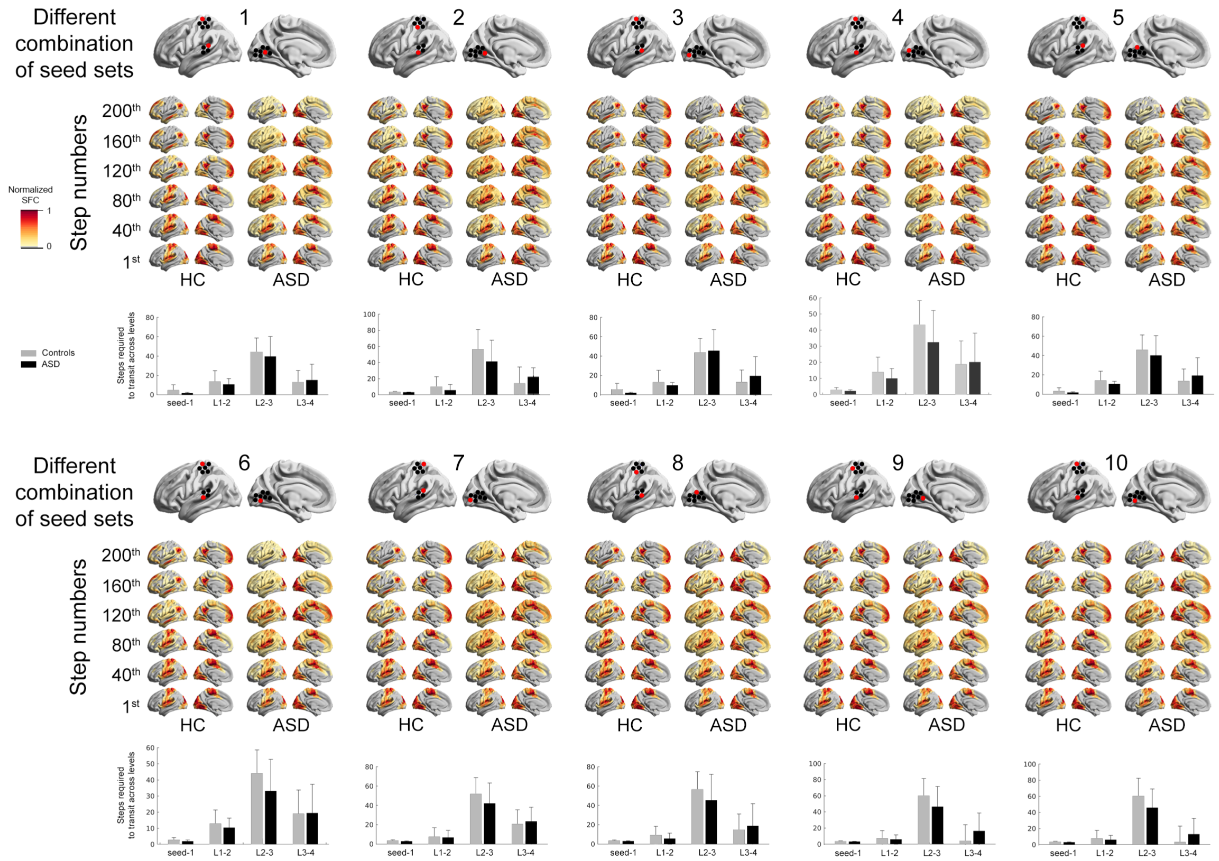

SUPPLEMENTARY FIGURE 11. Consistency of stepwise functional connectivity (SFC) findings when varying the coordinates of the seeds within primary sensory areas (10 iterations, V1, A1, S1). In all examples, one can observe an incomplete SFC propagation towards the transmodal core (situated in hierarchy level 4) in ASD. For further details, see ***Fig. 2.*** Error bars in the graphs represent 1 standard deviation.

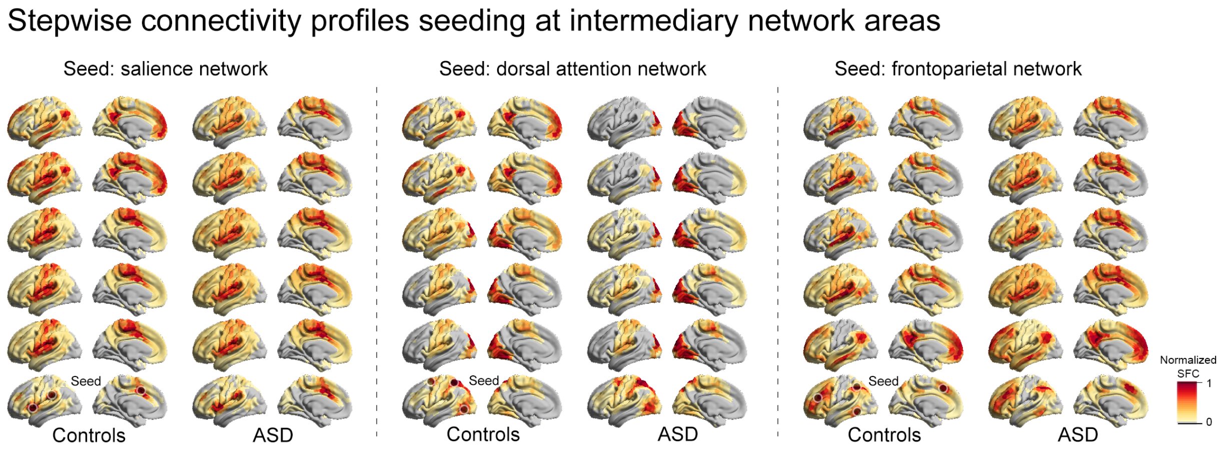

SUPPLEMENTARY FIGURE 12. Consistency of stepwise functional connectivity (SFC) findings when placing the coordinates of the starting seeds into intermediary networks (Salience, Dorsal Attention, Frontoparietal Network). In all examples, one can observe an incomplete SFC propagation towards the transmodal core in ASD. For further details on the procedures, see *Figure 2*.

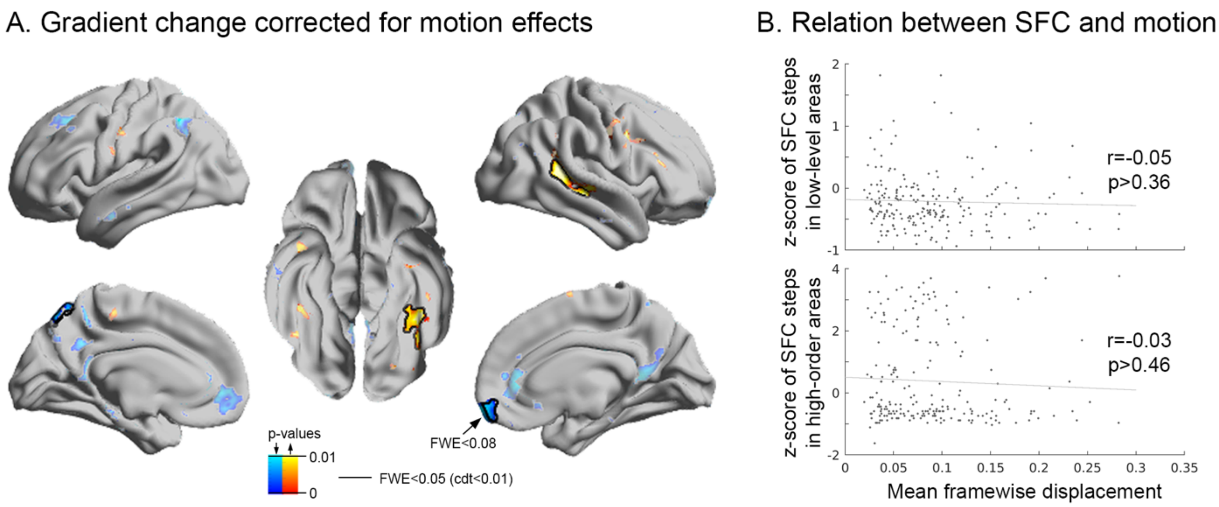

Supplementary Figure 13. A) Consistency of between-group differences in the first gradient scores after additionally regressing out mean frame-wise displacement (FD). For details on the statistical procedures, see *Figure 1*. **B)** Weak Pearson correlation coefficient between stepwise functional connectivity (SFC) step length and FD suggest low likelihood for an association between findings and motion.

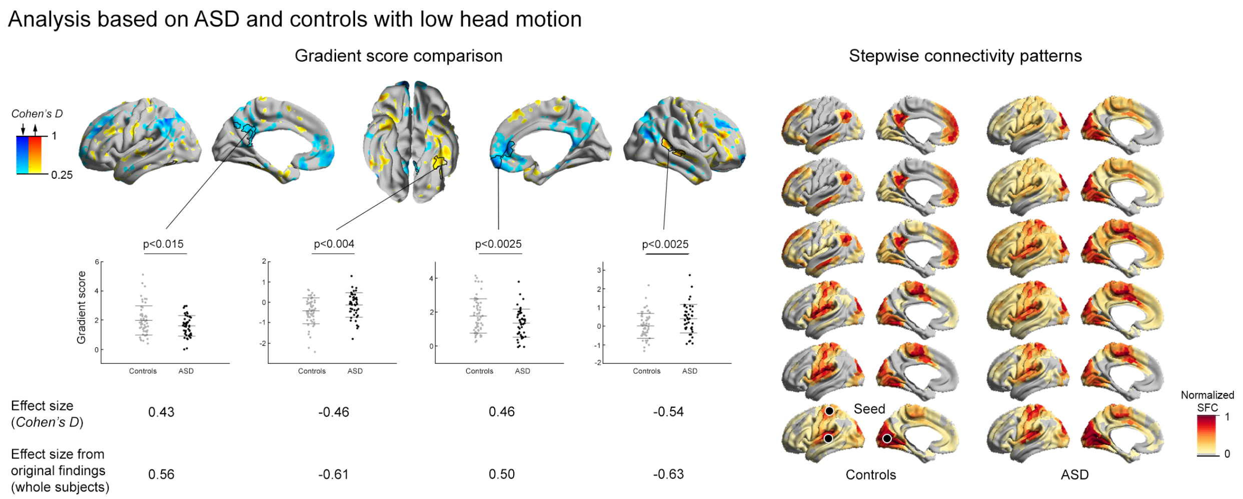

Supplementary Figure 14. Analysis based on ASD and controls with low head motion. *Left*. Effect size map (*Cohen's D*) from the comparison of gradient scores between ASD and healthy controls. Low head motion participants had a mean framewise displacement (FD) below the median FD of entire sample. In the graph below, the distribution of individual gradient scores averaged in each significant cluster is shown in both groups. Effect size of the analysis in the low head motion group is presented next to that of the original analysis based on entire cohort. Clusters were derived from the original group comparison (see **Figure 1**). *Right*. Stepwise connectivity patterns in the low head motion group. For details, see *Figure 2*.

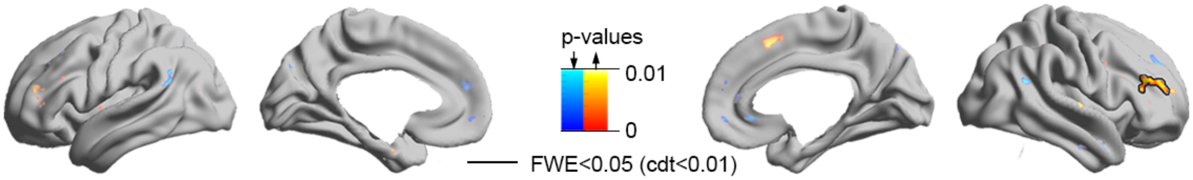

Supplementary Figure 15. Surface-based comparisons in gradient scores between control participants that showed low head motion versus those showing high motion. Statistical procedures are adapted from *Figure 1*. Comparing high and low movers does not recapitulate the ASD vs control differences in gradient findings.
