# Supplementary material for: Atypical functional connectome hierarchy in autism
Source: Nat Commun. 2019 Mar 4;10:1022. doi: 10.1038/s41467-019-08944-1 (PMC6399265; doi:10.1038/s41467-019-08944-1)
Supplement: Supplementary file 5 — Description of Additional Supplementary Files [file 41467_2019_8944_MOESM5_ESM.docx]

Description of Additional Supplementary Files

**SUPPLEMENTARY MOVIE 1**. Stepwise functional connectivity analysis, seeding from V1 in healthy controls (left) and autism (right). The video is uploaded as a separate file (SUPPLEMENTARY MOVIE 1.mov).

**SUPPLEMENTARY MOVIE 2**. Stepwise functional connectivity analysis, seeding from A1 in healthy controls (left) and autism (right). The video is uploaded as a separate file (SUPPLEMENTARY MOVIE 2.mov).

**SUPPLEMENTARY MOVIE 3**. Stepwise functional connectivity analysis, seeding from S1 in healthy controls (left) and autism (right). The video is uploaded as a separate file (SUPPLEMENTARY MOVIE 3.mov)
